# Open-Ring Enhancement in Pseudotumoral Multiple Sclerosis: Important Radiological Aspect

**DOI:** 10.1155/2014/951690

**Published:** 2014-04-15

**Authors:** Frederico Carvalho de Medeiros, Lucas Alverne Freitas de Albuquerque, Jose Eymard Homem Pittella, Renata Brant de Souza, Antonio Pereira Gomes Neto, Paulo Pereira Christo

**Affiliations:** ^1^Department of Neurology, Santa Casa de Belo Horizonte, MG, Brazil; ^2^Department of Neurosurgery, Santa Casa de Belo Horizonte, MG, Brazil; ^3^Pathology Service, Clínics Hospital, Faculty of Medicine of Ribeirão Preto, University of São Paulo, Ribeirão Preto, SP, Brazil

## Abstract

*Introduction*. Observation of open-ring enhancement in magnetic resonance imaging (MRI) is considered a specificity marker for diagnosing pseudotumoral multiple sclerosis (MS). This finding is of great value in the differential diagnosis of tumefactive lesions. *Case Report*. We describe a 55-year-old white woman, with previous history of ovarian cancer and recent history of fatigue and bilateral retroorbital pain. Important bilateral visual impairment evolved over one month. Physical examination detected the presence of right homonymous hemianopia. Cranial MRI showed an expanding lesion with open-ring enhancement. Given the range of diagnostic possibilities, a stereotactic biopsy was performed, and histopathological examination was consistent with an active demyelinating disease. The patient was treated with 1 g of methylprednisolone and symptoms improved following a significant reduction in the lesion. *Conclusions*. We highlight the MRI results suggestive of pseudotumoral MS, especially open-ring enhancement, which is an important radiologic aspect to diagnosis and can assist in avoiding unnecessary biopsies.

## 1. Introduction

Pseudotumoral multiple sclerosis (MS) is a diagnostic challenge, particularly when the patient's medical history is incompatible with MS [1]. Its prevalence is estimated to be 1-2 cases per 1000 cases of MS [1, 2]. There seems to be no predominance between genders; greater frequency is reported in patients aged 10–30 [1]. The MRI is characterized by a single lesion, larger than 2 cm, showing a mass effect, perilesional edema, and ring enhancement when using contrast [3]. This atypical form of MS mimics other tumor-like lesions, including neoplasms (gliomas, lymphomas), infections (abscesses, parasitic cysts), and infarction [1, 3]. This paper discusses the importance of careful radiological study in the differential diagnosis, particularly open-ring enhancement, whose presence alerted us to the possibility of demyelination, confirmed by biopsy.

## 2. Case Report 

**Figure 1:**
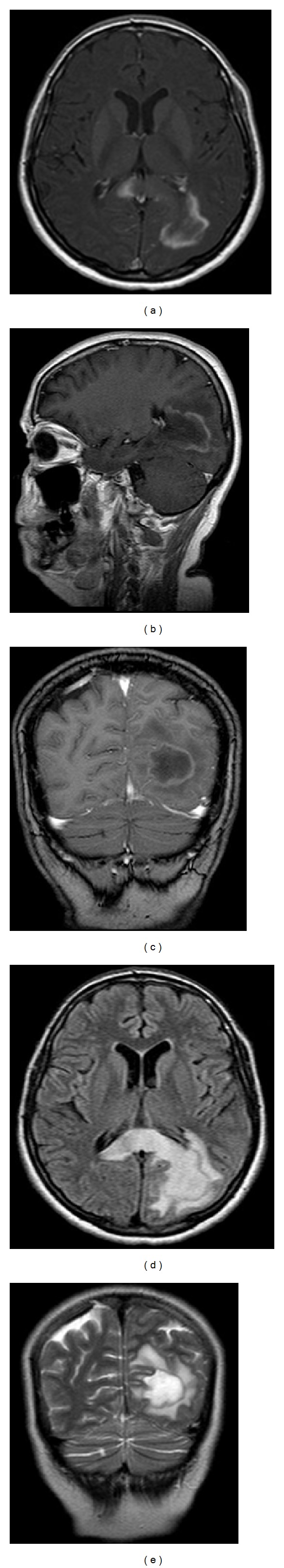
Cranial MRI showing (a) axial, (b) sagittal, and (c) coronal T1-weighted contrast enhanced views with open-ring enhancement of a hypointense lesion in the parietooccipital region with splenium corpus callosum involvement. (d) Axial FLAIR-weighted image showing hyperintense lesion in the parietooccipital region with extensive splenium corpus callosum involvement. (e) Rim lesions: coronal T2-weighted images showing a hypointensity margin relative to the hyperintensity of the lesion center and periphery.

A 55-year-old white woman with previous history of ovarian cancer and no recent history of vaccinations or infections reported fatigue and discrete bilateral retroorbital pain. Over the following month, her condition evolved, with important visual impairment in the right eye and later in the left eye. A physical examination revealed homonymous hemianopia D and tetrahyperreflexia. A cranial MRI (Figure 1) showed an expansive left temporooccipital lesion extending to the splenium of the corpus callosum, which measured 3.7 × 2.4 × 1.8 cm along its largest axes. Peripheral contrast uptake revealed an open-ring format, which was hypointense in T1-weighted MRI and hyperintense in T2 and FLAIR, and presented significant vasogenic edema of the adjacent white matter and unrestricted diffusion.

**Figure 2:**
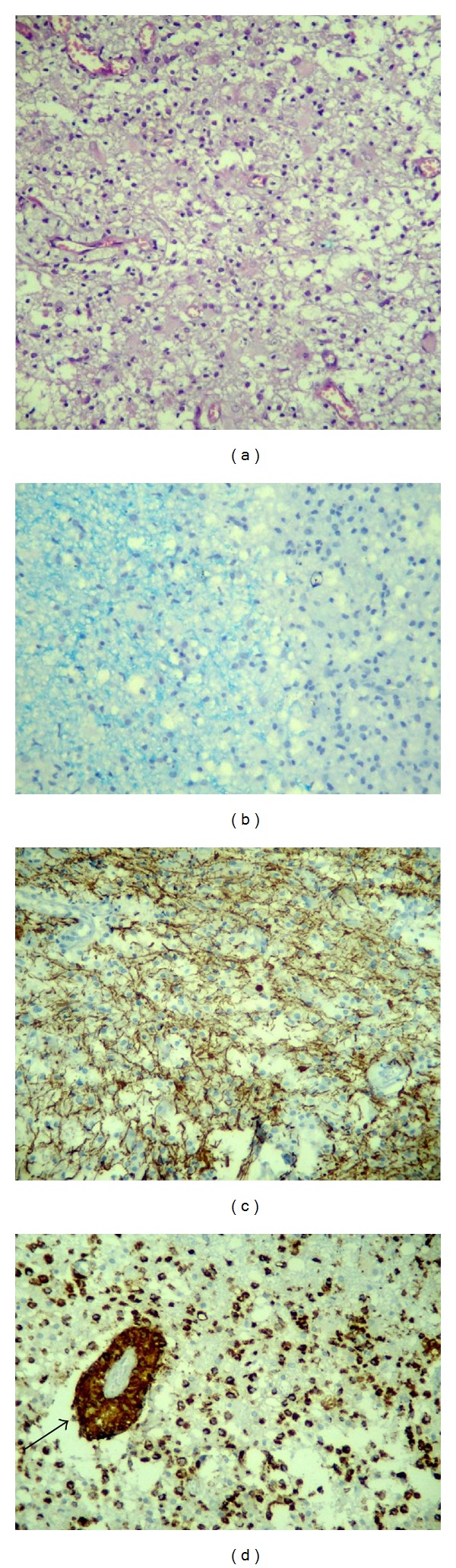
Histological sections of cerebral white matter of pseudotumoral multiple sclerosis. (a) Increased cellularity of the white matter due to infiltration by macrophages. Hematoxylin-eosin. (b) Junction of the normal (left) and demyelinated white matter (right), showing the well-defined limits of the demyelinated area. Luxol fast blue. (c) Relative axonal preservation in the demyelinated area. Immunohistochemistry for neurofilament. (d) Diffuse and perivascular infiltration (arrow) by macrophages. Immunohistochemistry for CD68.

Given the possibility of a primary tumor (glioblastoma, lymphoma), metastasis (previous history of ovarian cancer), or infectious, inflammatory, or pseudotumoral demyelinating process, a stereotactic biopsy was performed (Figure 2).

Histological sections stained with hematoxylin-eosin and luxol fast blue (for myelin) and immunohistochemistry for neurofilament and CD68, markers used to identify axons and macrophages, respectively, showed well-demarcated areas of demyelination in the white matter. A reduction in the number of oligodendrocytes, relative axonal preservation, diffuse infiltration by foamy macrophages (also identified around the vessels), mild perivascular lymphocytic inflammatory infiltration, and the proliferation of reactive gemistocytic astrocytes were observed permeating and surrounding the lesion. Neither intranuclear inclusions in the oligodendrocytes nor reactive astrocytes with hyperchromatic and pleomorphic nuclei were observed. The histopathological findings were consistent with an active demyelinating disease.

Inflammatory and rheumatologic tests were negative. Cerebrospinal fluid showed one cell, 46 mg/dL proteins, the absence of oligoclonal bands, and negative oncotic cytology.

**Figure 3:**
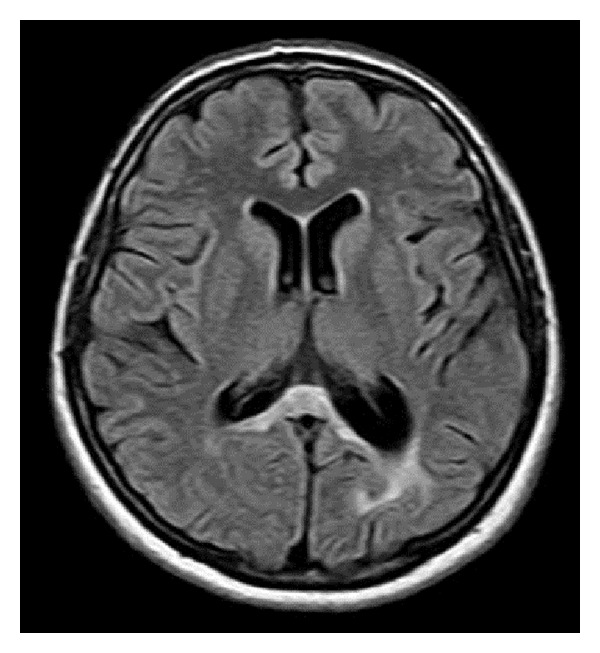
FLAIR-weighted cranial MRI demonstrating important reduction in the lesion following treatment.

Treatment involving pulse therapy with 1 g methylprednisolone for five days showed improvement in visual acuity. After six months, a cranial MRI revealed a significant reduction in the lesion (Figure 3).

**Figure 4:**
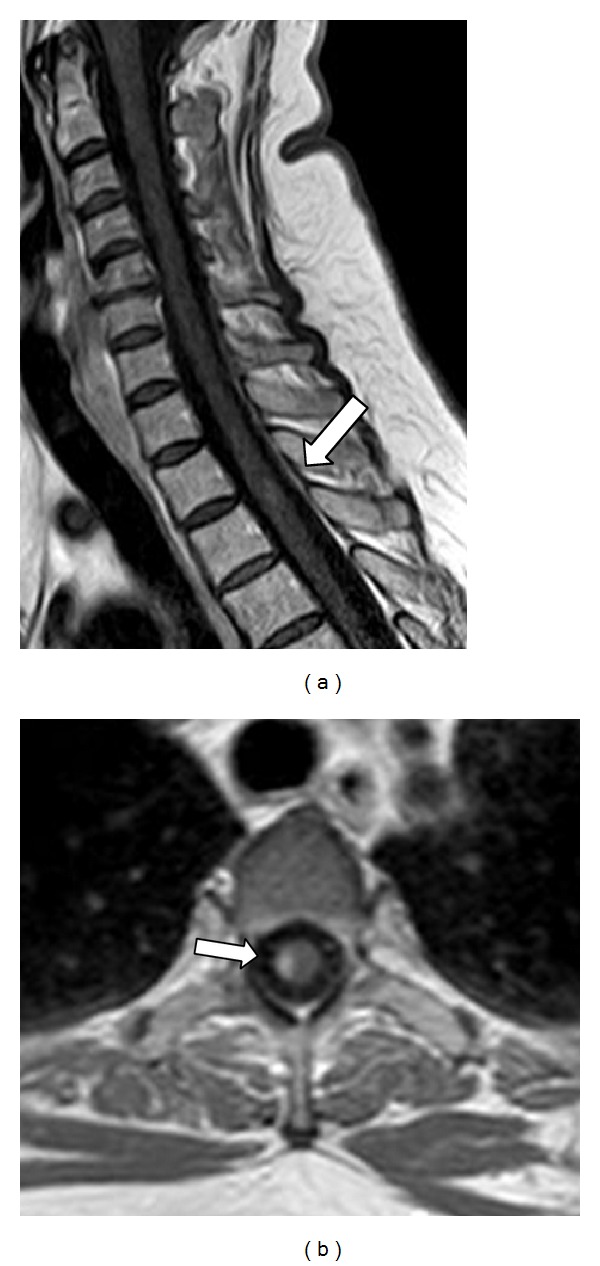
(a) Sagittal and (b) axial T1-weighted cervical/thoracic MRI showing contrast enhanced lesion at the T2-T3 level.

One month after the first symptoms, the patient presented paresthesia in all four limbs and mild paresis of the right leg. A gadolinium-enhanced T1-weighted cervical/thoracic MRI (Figure 4) showed a lesion at the T2-T3 level. The patient was submitted to additional pulse therapy with 1 g of methylprednisolone for five days and achieved complete remission of all symptoms. In a total of three years of follow-up, there were no other relapses and the patient is currently asymptomatic.

## 3. Discussion

In contrast to neoplasia and infections, ring enhancement of demyelinating lesions is frequently open, with the incomplete portion directed toward the cortex or the basal ganglia [4, 5]. The enhanced area resembles a crescent that only involves the white matter [5]. The highlighted region seems to represent the area of active inflammation, while the unenhanced area represents the inflammatory process in its most chronic phase [1, 4].

Masdeu et al. evaluated the specificity of open-ring enhancement in atypical demyelinating lesions. All the images were analyzed twice by two different independent neuroradiologists [5]. The specificity of the first neuroradiologist was 93.8 (95% confidence interval [CI] = 86–98) and of the second was 84.4 (95% CI = 74–92) [5]. Although this characteristic is highly specific for atypical demyelination, other more common lesions, such as tumors and infections, can present very similar patterns on rare occasions [5]. Typically, ring enhancement is complete in abscesses and cancer; however, in the former, it is smooth and thin, while in the latter, it is thick and irregular [3].

Other strongly suggestive features of demyelination in MRI include discrete mass effect or vasogenic edema; dilated vessels in the center of the lesion in T2-weigthed MRI image; marked reduction in blood flow perfusion compared with normal white matter, which flows slower than in abscesses, lymphomas, and high-grade gliomas; and rapid resolution of the lesion following treatment with corticosteroids, which can also occur in lymphomas [1, 4, 6]. Another typical feature of demyelination includes iso- or hypointense signal in T2-weigthed MRI image along the lesion margins [1, 7] (Figure 1(e)). Involvement of the corpus callosum and spectroscopy showing a diminished peak of N-acetyl aspartate and high levels of lipids, choline and glutamate/glutamine, and increased diffusion coefficients are nonspecific findings, which are primarily observed in glioblastomas and in lymphomas [1, 4, 6].

In our case, we observed just a moderate edema besides the extensive brain lesion, what we attribute to the subacute phase (one month of evolution). The slight swelling and mass effect related to pseudotumoral MS were previously reported by other authors [1]; they are even slighter in smaller or more chronic lesions [1].

Our patient presented some radiological characteristics suggestive of neoplasia. Despite the risk of metastasis because of the previous history of ovarian cancer (metastases do not possess a typical radiological pattern), the radiological characteristics were primarily those of high-grade glioma: an expansive lesion with important vasogenic edema and involvement of the corpus callosum that exceeded the midline. The only radiological data suggestive of pseudotumoral demyelinating disease was open-ring enhancement. Furthermore, the negative CSF analysis for oligoclonal bands (positive in up to 30% of cases of pseudotumoral MS), the clinical presentation limited to visual impairment (compatible with the lesion location), and the absence of multiple lesions in the MRI at the time of the biopsy (present in up to 70% of patients with pseudotumoral MS) make this a rare and challenging case [8]. Due to the heightened suspicion of neoplasia, the doctors opted to perform a stereotactic biopsy.

Histopathological examination identified changes that favored a diagnosis of an active demyelinating lesion, since it demonstrated the products of the breakdown of the myelin sheath inside foamy macrophages, visible as blue granules in luxol fast blue staining. Macrophages were aligned with preserved axons, as determined by immunohistochemical staining for CD68 and neurofilament, respectively. Perivascular chronic inflammatory infiltration of lymphocytes and macrophages in the absence of necrosis was indicated by hematoxylin-eosin staining and typical reactive gemistocytic astrocytes were distributed between and around the lesion [9, 10].

Caroli et al. affirmed that here are two determinants for error in histological diagnosis: biopsies performed in areas of intense gliosis, with atypical astrocytes, favor a misdiagnosis of glioma [11]. Moreover, tissue samples from the center of the lesion, due to the presence of a large number of macrophages, can be misdiagnosed as cerebral infarction, unless staining for myelin and axon is performed [11].

Recent studies have established a correlation between certain MRI findings and the results of the histopathological examination: peripheral hypointensity in T2-weigthed MRI image corresponds to myelin degradation and the infiltration of macrophages; the intensity of peripheral enhancement correlates with the degree of perivascular infiltration of these cells; and perilesional, vasogenic, and hyperintense edema in T2 reflect increased permeability around the lesion [7].

The evolution of this case confirmed the histopathological diagnosis, since the patient responded well to pulse therapy, and despite relapse disease in the thoracic cord, she responded well to the second course of steroids. According to some studies, one-third (1/3) to two-thirds (2/3) of those who initially develop a tumefactive MS lesion progress to the recurrent, remittent course typical of MS [1, 2].

A definitive diagnosis of pseudotumoral MS is a major challenge. In the spectrum of single tumefactive lesions, neurologists, neurosurgeons, neuroradiologists, and neuropathologists should always consider MS in the differential diagnosis. Careful radiological study, directed towards specific findings for demyelinating lesions, mainly open-ring enhancement, could assist in avoiding invasive procedures that are not risk-free, such as stereotactic biopsy. This case demonstrated that even in a patient with a significant risk of metastatic lesion, the radiological aspects were in accord with the histological confirmation of pseudotumoral MS.

However this was a single and illustrative case and more research is required to assess the risks and the benefits for the patient in establishing a management approach without histopathological confirmation, when analyzing a suggestive image of a disease that is more common and severe in daily practice, such as high-grade gliomas.

## References

[1] Hardy TA, Chataway J (2013). Tumefactive demyelination: an approach to diagnosis and management. *Journal of Neurology, Neurosurgery & Psychiatry*.

[2] Turatti M, Gajofatto A, Bianchi MR, Ferrari S, Monaco S, Benedetti MD (2013). Benign course of tumour-like multiple sclerosis. Report of five cases and literature review. *Journal of the Neurological Sciences*.

[3] Javalkar V, Manix M, Wilson J, Nanda A (2012). Open ring enhancement in atypical brain demyelination. *Journal of Clinical Neuroscience*.

[4] Given CA, Stevens BS, Lee C (2004). The MRI appearance of tumefactive demyelinating lesions. *American Journal of Roentgenology*.

[5] Masdeu JC, Quinto C, Olivera C, Tenner M, Leslie D, Visintainer P (2000). Open-ring imaging sign: highly specific for atypical brain demyelination. *Neurology*.

[6] Akimoto J, Nakajima N, Saida A, Haraoka J, Kudo M (2006). Monofocal acute inflammatory demyelination manifesting as open ring sign. *Neurologia Medico-Chirurgica*.

[7] Kobayashi M, Ono Y, Shibata N (2009). Correlation between magnetic resonance imaging findings and pathological observations in tumefactive multiple sclerosis. *The Neuroradiology Journal*.

[8] Rahmlow MR, Kantarci O (2013). Fulminant demyelinating disease. *The Neurohospitalist*.

[9] Sugita Y, Terasaki M, Shigemori M, Sakata K, Morimatsu M (2001). Acute focal demyelinating disease simulating brain tumors: histopathologic guidelines for an accurate diagnosis. *Neuropathology*.

[10] Neelima R, Krishnakumar K, Nair MD (2012). Tumefactive demyelinating lesions: a Clinicopathological correlative study. *Indian Journal of Pathology and Microbiology*.

[11] Caroli E, Salvati M, Ferrante L (2006). Tumor-like multiple sclerosis: report of four cases and literature review. *Tumori*.

